# Older adults' acceptability and perceived barriers to digital health tools integrating nutrition and physical activity: a focus group study

**DOI:** 10.3389/fdgth.2026.1803847

**Published:** 2026-04-22

**Authors:** Gioi Spinello, Erica Gobbi, Antonio Paoli, Tatiana Moro

**Affiliations:** Department of Biomedical Sciences, Laboratory of Nutrition and Exercise Science, University of Padova, Padova, Italy

**Keywords:** diet, eHealth, elderly, exercise, focus group, mHealth

## Abstract

**Introduction:**

The rapid ageing of the population presents significant public health challenges, particularly in countries with high life expectancy such as Italy. Although nutrition and physical activity are key determinants of healthy ageing, many older adults do not meet recommended guidelines. Mobile health (mHealth) technologies may support healthy behaviors; however, evidence on older adults' perspectives remains limited, especially in the Italian context. This study aimed to explore experiences, perceptions, and expectations regarding mHealth tools for nutrition and physical activity.

**Methods:**

A total of three in-person focus groups were conducted with older adults in Italy, recruited regardless of prior experience with mobile health technologies. Data were analyzed using reflexive thematic analysis.

**Results:**

Reflexive thematic analysis generated three themes: digital health as a “robotic friend”, digital health as an emotional barrier and digital health to increase awareness. The findings demonstrated that participants had good mHealth literacy. Nevertheless, they described digital health technologies as low in engagement and external motivation, highlighting the emotional distance, a strong preference for in-person interactions, and a general mistrust toward digital health. While there were some concerns related to privacy and fear of injury, older adults expressed their interest in digital tools as sources of guidance, education, and supervision

**Discussion:**

Fully automated digital interventions may not meet the needs and preferences of this population. Findings suggest that hybrid models combining both digital technologies and human interaction might be more acceptable and feasible for promoting physical activity and healthy nutrition in later life.

## 1 Introduction

The population of older adults is increasing rapidly, and by 2050, the number of people aged 60 years and over is projected to double to 2.1 billion worldwide (1). Italy, in particular, is recognized as one of the top countries worldwide in terms of life expectancy. In 2021, life expectancy at birth in Italy was 82.2 years compared with 76.3 years in Europe and 71.4 years globally (2).

However, healthy life expectancy, the average number of years lived in good health, stands at 70.6 years in Italy, compared with 66 years in Europe and 61.6 years worldwide, indicating that individuals spend approximately 10 years of their life in poor health (2).

The interaction between the persistent decline in births and high life expectancy has determined a demographic transformation in Italy, with an increase in the average age of the population. This demographic change raises concerns about the burden of an aging society in poor health and affected by chronic diseases (3). A considerable proportion of this burden is linked to behavioral risk factors, many of which are preventable through greater awareness and effective public health policies (3).

Lifestyle plays a crucial role in healthy aging (4, 5), with nutrition and physical activity being two of the most influential factors for maintaining health and independence in later life (6, 7). The benefits of healthy nutrition (8, 9) and regular physical activity (10, 11) are well documented; however, most older adults do not meet the recommended guidelines for either (12, 13).

Physical activity is a key determinant of cardiovascular health, muscle strength, mobility, and cognitive function (11), while adequate macronutrient and micronutrient intake prevents malnutrition and sarcopenia (9). Together, these lifestyle factors help to sustain a high quality of life and mitigate the risk of functional decline and frailty.

The upcoming goal and challenge of societies should be to promote successful aging by developing specific health intervention strategies addressed to the growing population of people aged 50 years and above. When properly designed and implemented, digital health technologies (eHealth), may offer valuable tools to encourage healthy behaviors and extend years lived in good health. Research has shown that eHealth interventions encourage and stimulate health behavioral changes by offering tailored information and strategies such as goal-setting and continuous monitoring (14–16). Moreover, their relatively low cost can potentially reach a large number of individuals (17, 18).

Despite these advantages, older adults tend to be less inclined to use eHealth tools (19, 20). Nevertheless, we are facing a transition period with digital literacy increasing among older population. In Italy, 68.1% of people aged 65–74, and 31.4% of people older than 75 years, connected to the internet at least once in 2024. Compared with 2023, internet use increased by 7.6% among people between 65 and 74 years and 6.7% among people older than 75 years (21).

Even with this growth, older adults tend to remain behind younger generations in embracing technology. Therefore, considering the rise of the older Italian population and the possibilities that eHealth can offer, it is essential to understand their perceptions, experiences, and suggestions regarding such tools, particularly for lifestyle habits with a major impact on health, such as nutrition and physical activity, which can be supported through smartphone applications.

While most research has focused on the effectiveness and efficacy of digital technology in promoting physical activity and healthy eating, fewer studies have explored older adults' perspectives. To our knowledge, no previous research has investigated this topic in the Italian older population. Therefore, the purpose of this study was to explore the perspectives and opinions on mobile health to design accessible, feasible, and impactful apps to support healthy habits and lifestyle of older population.

## 2 Materials and methods

### 2.1 Participants

Participants were recruited through a senior cultural association in Padua, using digital and printed materials and word-of-mouth. To be eligible, participants should be aged 65 years old or older, able to live independently and able to understand and follow instructions.

Overall, participants were aged between 66 and 74 years old (9 women and 6 men). Three out of fifteen participants were overweight or obese, while the remaining participants had a normal body mass index. Six participants reported hypertension, three reported impaired glucose tolerance or type 2 diabetes, and three reported hypercholesterolemia.

With regard to physical activity, six participants reported not being currently involved in structured physical activity, while the others reported participating in organized exercise activities. Detailed participants' characteristics are presented in Table 1.

**Table 1:** Participants' characteristics.

Focus group ID	Number of participants	Sex	Age (years)	Level of education^a^	Principal occupation	Previous experience with mobile phone applications
Focus group 1	6	2M, 4W	70.7 ± 1.6	– Certificate (1) – Diploma (3) – Bachelor's degree (2)	– Office worker (3) – Human resources and hiring manager – Housewife – Surveyor	100%
Focus group 2	5	2M, 3W	69.6 ± 4.2	– Certificate (2) – Diploma (3)	– Mortgage broker – Office worker (2) – Storekeeper – Computer consultant	100%
Focus group 3	4	2M, 2W	69.5 ± 3.3	– Certificate (1) – Diploma (2) – Master's degree (1)	– Salesman – Lawyer – Office worker (2)	100%

M = men, W = women.

a Classification of “level of education” was based on the highest level of education completed at the time of data collection.

Participants were recruited regardless of their prior experience using smartphone applications or other digital health platforms related to nutrition or physical exercise. This approach allowed us to capture a broader and unbiased range of perceptions regarding the use of digital health technology to support healthy aging. However, it is important to acknowledge that communication within the association usually occurs through mailing lists and digital announcements. As a result, participants may have had a relatively higher familiarity with digital communication tools compared with the general older adult population.

We conducted three focus groups from September 2024 to December 2024.

Before taking part in the study, they were provided with detailed information regarding the study's aims and procedures, and they were required to read and sign informed consent. Participants were informed that the general aim of the study was to explore older adults' perspectives, experiences and feelings regarding digital health technologies to support healthy ageing, without presenting specific hypotheses in order to minimize expectancy effects. The study was approved by the Internal Review Board of the Department of Biomedical Sciences of the University of Padua (HEC-DSB/2-2024).

### 2.2 Data collection

Before conducting the focus group, a multidisciplinary team composed of two psychologists, three sport scientists, and a nutritionist developed a semi-structured question protocol to ensure consistency and direction in the discussion, stimulating relevant responses without limiting the participants' spontaneity (Supplementary Material S1). The question guide was conceived as a set of core prompts, which were asked in all focus groups to allow comparison across groups. These core questions were accompanied by example probes and follow-up questions adapted to issues raised spontaneously by participants. All focus groups were moderated by the first author (G.S.), a female PhD candidate in sport sciences under the age of 30. She had an academic interest in digital health technologies to support healthy ageing. In line with qualitative research reflexivity, these assumptions and potential influences were acknowledged and considered during the analytic process. To minimize undue influence and leading questions during data collection, the moderator used neutral, non-evaluative language, avoided expressing personal opinions, and encouraged all participants to speak.

All focus groups were performed in Italian and took place in person in the Department of Biomedical Sciences, Nutrition and Exercise Science Laboratory. Each session lasted from 55 to 65 minutes, was audio- and video-recorded, and later transcribed manually by G.S. No previous relationship existed between the moderator and the participants before the study. At the beginning of each focus group the moderator presented herself and explained the aim of the study for the second time, the first explanation was provided when participants read and signed the informed consent. Participants were then invited to introduce themselves to encourage open discussion. They were reminded that there were no wrong or right answers and that the aim of the study was to collect their personal experiences and expectations from an application related to nutrition and exercise.

### 2.3 Data analysis

Before starting the focus group, we collected sociodemographic information for each participant, including age, level of education, and previous profession (most participants were retired). After completing all three focus groups, data analysis began. The audiotapes were transcribed by G.S., and she used the video recording to note non-verbal communication. When transcribing, all participants were assigned identification codes to protect their privacy. Interview transcripts were uploaded into the NVivo qualitative analysis software (QSR NVivo analysis software 12.2). For data analysis, we decided to use a reflexive thematic analysis approach (22, 23). After familiarizing herself with the transcripts through repeated reading, the first author began noting initial impressions and observations. Preliminary codes were then generated from the semantic content of the data. Once the entire dataset had been coded, the codes were examined and organized to identify broader patterns of meaning, leading to the development of themes and subthemes. These themes were subsequently reviewed, refined, and clearly defined. In accordance with the chosen reflexive thematic analysis approach, coding and theme development were conducted by a single researcher, with the inherently interpretative and iterative nature of this process made explicit to enhance transparency.

When reporting the findings, data extracts were presented with interpretations contextualized in relation to existing literature, as recommended by Braun and Clarke (24) and Terry and colleagues (25). In line with reflexive thematic analysis, themes were not pre-decided but were actively generated, with codes and themes evolving during the analysis.

## 3 Results

### 3.1 Overview

This study aimed to better understand perspectives and feelings of older adults regarding the use of digital health for nutrition and exercise. Fifteen older adults (mean age 70.0 ± 2.9 years) participated in the three focus groups. Participants reported different experiences and levels of familiarity with mHealth technologies related to both physical activity and nutrition. All participants stated having used a step counter at least once in their lifetime, either on their smartphone or through a smartwatch connected to their phone. They mentioned tracking steps, calories burned, distance covered, and, in some cases, elevation changes during hikes.

Four female participants reported using video tutorials on YouTube during the COVID-19 lockdown to stay active. Three others continued their habitual physical activity online with virtual classes organized by their trainers. One woman used an app for yoga during lockdown, while a male participant used an app focused on abdominal workouts.

Regarding the relationship between nutrition and eHealth, only one participant had used a calorie-counting app. However, she abandoned it because the most interesting and useful features required payment. Fourteen out of fifteen participants said that they never used a caloric-tracking app, and only one participant was unaware that such apps existed.

We purposefully recruited participants from different backgrounds without explicitly requiring prior experience with mHealth for nutrition or physical activity. Nevertheless, we observed mixed levels of familiarity with the apps on their smartphones.

During the three focus groups, different themes emerged, particularly concerning participants' feelings about using digital health tools for physical activity and nutrition. As typical of qualitative research, the findings are context-specific and intended to provide in-depth insights rather than statistical generalizability.

### 3.2 Digital health, a robotic friend

Emotional detachment emerged as a recurring theme associated with apps. Participants perceived apps as cold and uninterested in their experience; moreover, interacting with them felt distant and impersonal. Considering the non-human entity of this technology, participants find interacting with it anxiety-inducing. As one participant remarked:

“It is a metallic voice that tells you what to and not to do, absolutely no, I don't like it!”

Continuous self-tracking and frequent notifications were frequently perceived as oppressive, generating anxiety rather than empowerment. One woman mentioned that her daughter successfully used a calorie-tracking app, but, reporting her words:

“I think she can do it because she is an engineer.”

All participants (15/15) expressed a clear preference for in-person care. Those who used apps during the COVID-19 lockdown (7 out of 15) reported deleting them shortly after in-person activities were resumed. This pattern has also been observed in other studies (26, 27). Participants who engaged with video tutorials for physical exercise affirmed that their use was driven by the context of the pandemic and viewed as situational and that they would only consider using such tools again in case of another lockdown. Three stated they would never use an exercise app again under any circumstances. These findings reflect patterns reported in other studies, where digital adoption during the pandemic did not translate into long-term adherence (28, 29).

Participants who decided not to engage in online activities explained that they considered physical activity a social experience and an opportunity to spend time with friends. One participant noted:

“I go to the gym classes because being in person with others gives me much more satisfaction. Doing it alone does not give me that satisfaction, even if I have to go outside. And this is important for me.”

Not surprisingly, most participants preferred group training and in-person support, whether for exercise or nutritional advice. Different psychological and social theories emphasize that social interactions are a core aspect of the individual's needs. According to Self-Determination Theory (30), basic psychological needs are competence, autonomy and relatedness, and technology fails to support them. As people age and social networks inevitably diminish, group training and opportunities for in-person interaction may strengthen a sense of belonging and reduce isolation (30, 31). These findings align with previous studies (28, 29, 32) showing that older adults prefer in-person classes over web-based physical activity interventions.

Moreover, when referring to intervention generally, motivation arises as a key aspect, especially when the goal is to change habits and increase exercise or diet adherence. Intrinsic motivation is not easy to achieve; social support often plays a crucial role. In the last focus group, this tension between personal motivation and the need for external social support was a theme discussed:

E: “Well the problem is always the same, it must start from us … If I decide to take the path in this app. I do everything, I put data in … and everything … don't know if sending messages may help.”
G: “Well maybe it could give the motivation to carry on with what we are doing.”
E: “Yes for sure … but, you see I need to discipline myself and my problem is often the lack of discipline.”
G: “Believe me, not just yours.”
F: “Well me too, I like eating and spending the day on the sofa: lack of discipline is my problem too.”
L: “Okay but if you decide to commit, you have to…”
F: “Of course, the point is that the app doesn't fit because having a person in front of you is motivational … the app is not motivational, unfortunately.”
E: “If you have to manage yourself you do not exercise, while if you have a commitment with another person you do it!”
L: “Well but this is a lifestyle choice…”
F: “Of course but having a person is a sort of help, I easily try to avoid doing effort. I need someone to … to help me maintain motivation. There are those who are more determined, but that's definitely not my case.”

This discussion highlights a clear need for external motivational support, particularly in the form of social support, which participants felt current apps could not adequately replicate. Face-to-face interventions were consistently seen as more effective in sustaining engagement. However, research comparing the adherence between traditional in-person training and app-based interventions shows that digital tools can maintain motivation when designed with features, such as social support, reminders, educational content, and the flexibility of training timing (33–36).

In this sense, the focus groups highlighted the perceived novelty and potential value of a hybrid approach. Participants emphasized the need for solutions that integrate the standardization of digital applications with personalized, human input. Such an approach was described as a way to move beyond the limitations of current apps, towards a more tailored and interactive system, almost resembling telemedicine, in which individual data are interpreted by professionals to provide customized guidance. This combination of technological structure and human support was framed as a key element for enhancing both engagement and perceived effectiveness. This theme aligns with previous literature on digital health interventions and telemedicine, which suggests that hybrid models, combining standardized digital tools with ongoing personalized support, may be particularly beneficial for groups with lower intrinsic motivation or greater need for guidance, such as older adults (37, 38).

### 3.3 Digital health as an emotional barrier

Participants expressed skepticism about apps' capabilities to adapt to individual needs, considering that nutrition and physical activity require a high personalization. There was a general mistrust based on the idea that a standardized app cannot effectively address individual needs, given the significant variability in nutrition and physical activity requirements from person to person.

Moreover, they were worried about getting injured while using exercise apps, and they expressed concern about performing exercise not properly without supervision, which they felt could be counterproductive. One participant reported experiencing severe low back pain after using an app for abdominal workouts.

From the words of one participant referring to online physical activity:

“No, because I have always struggled, I have tried, but I struggle to repeat the exercise correctly if I don't see I cannot … and then if there isn't someone telling me if I'm doing it correctly, I am afraid of getting hurt…”

When referring to nutrition tracking, inserting dietary information was seen by participants as cumbersome, demanding, and even obsessive. The process of manual data entry was described as burdensome and monotonous. Moreover, many preferred to follow their own dietary habits rather than relying on structured digital tracking. Several participants felt that following rigid digital rules would require them to give up too much of what they enjoyed:

“If you tell me a list of things that I cannot eat and among them are twenty of my favorite foods, it is impossible for me to carry on.”

“Well, if I want to spend some time with my nephews, I always have sweets and junk food, I am not willing to give up my favorite food.”

“I imagine being at the restaurant and start typing what I eat … people are going to laugh at me.”

Among the older population, this reluctance to change is consistent with findings in the literature (39). However, a systematic review on nutrition app acceptance found that fitness apps are generally more accepted among middle-aged and older adults than other apps (40).

Concerns about trust and privacy were reported, and participants expressed discomfort about who might access their personal data:

“You know the name of the app, but you don't know who is behind that app.”

Others doubted the credibility of the information provided, pointing out the overwhelming and often contradictory nature of online information:

“On the Net you find everything, and in the end you cannot believe a thing … The only way is to experiment on yourself.”

Unreliability and mistrust of online health information is common among older adults, and previous studies have shown that older adults often perceive the internet as unreliable (41, 42).

Several participants emphasized that to use an app, one must first feel the need for it. Some questioned their usefulness:

“Why should I use an app? Nowadays you can find everything you want on the Net.”

“Nowadays, what you find online is easier to follow; you can even do a step-by-step recipe with a video on YouTube.”

Although these participants mostly rejected structured calorie-tracking apps, many demonstrated a relatively good eHealth literacy in other contexts. Ten participants (nine women and one man) reported using the internet or social media platforms (Instagram, Pinterest, YouTube) to search for recipes, whether to impress their guests, cook more balanced meals, or try something new. This suggests that while they do not lack eHealth literacy, they are more receptive to flexible, informal tools than to structured, prescriptive systems. Other studies have found that using apps to improve older adults' food awareness is feasible, but only if designed as adaptable and user-friendly (43, 44).

Participants' mixed attitudes toward online training further illustrate these feelings. Some participants who engaged in virtual training sessions during the COVID-19 lockdown found it challenging to follow instructions on a screen while exercising, and others reported losing interest as the online lessons continued. As one participant recalled:

“During COVID19 lockdown I did it, I tell you, but only with my trainer and at a certain point I said ‘Marina that's enough now, we can't take it anymore.’ But for a whole year we did it three times a week, always perfectly on time and I didn't miss a day because anyway it seemed right, she rightly saw me while I was exercising pointing out every possible error. So here it is, an app should have a person who … who gives me a stimulus like that, I need to have a person who speaks with me … Also, because I've tried and it's not easy to follow a person you don't know… you have to do and listen and you lose continuity but above all you lose contact, physical, visual and therefore for me it's mmmm…”

So initially online training was accepted however, as the months went on and curiosity faded, repetitive logging and less interaction with a real person felt monotonous and boring. This aligns with the systematic review by Perski and colleagues, who highlighted that digital engagement depends on affect, attention and interest, and that sustained use requires features that require a deeper involvement (45).

It is important to emphasize that participants in this study demonstrated decent eHealth literacy. Some also actively used social media, which can further strengthen digital competencies (46). However, the quality of online resources, especially on platforms, is not always reliable, and they may be particularly vulnerable to misinformation, as distinguishing credible from non-credible sources can be challenging. Regarding YouTube content, Rodriguez-Rodriquez et al. (47) reported that most exercise videos during lockdown were of low quality, while another study focusing on geriatric patients found that 78% of YouTube exercise videos were of moderate or low quality (48). Both studies highlighted the importance of checking the source of a video, recommending a preference for content produced by medical professionals or official institutions to avoid misinformation. It should be noted that these studies evaluated content in English, so results may not directly apply to Italian language content.

### 3.4 Digital health to increase awareness

During the focus groups, participants supported using digital health tools, particularly when integrated within a telemedicine framework. They envisioned an app that would provide personalized nutrition and exercise programs supported by regular follow-up appointments and a team of professionals. As discussed in the previous section, participants prefer a human interaction over an automated system; this reflects participants' need to understand the rationale behind recommendations, rather than simply receiving prescriptive instructions.

Regarding physical exercise, participants suggested that the app should offer a variety of weekly exercises, indicating which muscle groups were being targeted. They also suggested the inclusion of video tutorials with audio guidance during workouts to enhance engagement. As one participant stated:

“Well, definitely an app that gives you an explanation of why you are doing this or that. I don't like something that just tells me what to do and that's it! There should be a 'sign' that tells you why, for example, at a certain age someone may have high cholesterol, ok there is a genetic component, but you can still manage it. But it's difficult to find an app that does such a thing.”

Participants also proposed integrating the app into a structured model with tailored recommendations for specific conditions such as hypertension, diabetes or osteoarthritis. They were particularly interested in understanding beneficial food combinations and the interactions between diet, exercise and medications in relation to individual health conditions.

Reporting part of the dialogue that occurred during the first focus group:

A: “Yes, but for example, I would also like advice coming from certified sources. I mean, if there are real advances in medicine, professionals can change their recommendations accordingly. Over time, some things have been shown to make no sense, while others have been validated. For me, a notification with advice is fine, but it should come from certified sources—because nowadays you can find everything and anything on the Internet.”
(…)
B: “And in the end, you really stop believing in anything … you just end up experimenting on yourself, that's it … fingers crossed.”
A: “As I said before, when it comes to medications, I often feel like there's a lot of contradictory information—one thing says one thing, another says the opposite. So, if I receive a notification from the Institute of Physiology of Padua, for example, that would interest me. Getting information directly from a certified source is much more reliable than having to search randomly online for updates. I think that's very valuable.”
C: “Yes, ideally the app should let me enter my data—my conditions, the medications I take—and then provide tailored monitoring. That would really make a difference.”

The importance of learning in later life is well supported in literature. A systematic review and meta-analysis has shown that participation in lifelong learning contributes to increased quality of life, health, cognitive function, self-dependency, and sense of belonging (49). Moreover, evidence suggests that older adults can successfully learn with eHealth tools, particularly when are personalized (50, 51).

Nevertheless, high expectations towards digital health interventions sometimes fail to meet actual user experiences, leading to discouragement and eventual abandonment as described by Yardley and colleagues (52). Reporting the concerns of our participants:

“Well, I downloaded it, I started using it but if I wanted new features I had to pay so I abandoned it.”

“While I was on lockdown, I used it, but I know, when I am alone with no one following and correcting me, I am inconsistent … moreover, I suffered from such low back pain while I was using it. So I clearly stopped.”

Our analysis also revealed a limited awareness of the broader benefits of physical activity beyond calorie expenditure. Many participants framed exercise mainly in terms of burning calories, often in discouraging ways, as illustrated by the following quotes:

“Yes, I look at the steps and there is the matched calorie count and it is quite depressing already … In the sense that one takes 10,000 steps and you have not even earned a brioche!”

“Oh, sometimes I use it, obviously, I eliminated the calories because they were always too few. When I take many steps, I control them; when I take fewer, I take less time looking.”

These reflections suggest that while digital tools may increase awareness of energy balance, they may reinforce a narrow, calorie-focused perspective. A more comprehensive understanding of the role of physical activity in promoting wellbeing remains limited. However, these findings align with a recent systematic review, which observed that exercise is often perceived as a “license to eat” rather than an activity valued for enjoyment or health. Although this observation has primarily been reported in younger populations, our study suggests similar attitudes are also present among this subgroup of older adults.

Echoing Lupton, pushing people into self-tracking, can inadvertently impose new burdens and exacerbate inequalities (53).

## 4 Discussion

### 4.1 Limitations

While this study provides valuable context-specific insights, some limitations should be acknowledged. First, the sample size was relatively small. However, after three focus group interviews, the material was judged to contain sufficient information for analysis and thematic redundancy was observed across discussions. Second, participants were recruited from a senior cultural association in Padua and were independent and able to perform activities of daily living. Therefore, while the findings are not intended to be statistically generalizable, they may be transferable to similar contexts.

We also acknowledge that information on participants' socioeconomic status was not collected. As socioeconomic factors may influence access and familiarity with digital technologies (54), the absence of this data limits the possibility of further contextualizing the findings.

### 4.2 Interpretation and implications

The data collected across the three focus groups and the themes that emerged suggest that many older adults are increasingly engaging with digital health information and are narrowing the gap in digital health literacy. However, participants reported that their increased use of digital health tools during the COVID-19 pandemic was largely situational and did not translate into long-term adherence. In-person practice remains the preferred way to engage in physical activity, reflecting the value placed on social interaction and external support.

Participants also expressed limited awareness of age-specific nutritional needs and frequently rely on television, social media, and the internet as sources of dietary recommendations. These findings highlight the crucial role of society and public health services in providing reliable channels of health communication to minimize the risk of misinformation and support greater acceptance of digital tools among older adults.

Accordingly, several participants expressed a common feeling of mistrust toward digital health technologies. Apps were perceived as cold, impersonal, sometimes even anxiety-inducing, and many of them reported difficulties in sustaining motivation when using digital health tools, highlighting that external and social support are central for long-term engagement.

Taken together, these findings suggest that purely digital approaches may not adequately meet the needs of some older adults. Instead, participants highlighted the potential value of combining digital tools with opportunities for human interactions and social support. In this sense, hybrid approaches integrating digital resources with interpersonal guidance may represent a more acceptable and promising direction for supporting healthy aging.

Designing digital health technologies that are inclusive and responsive to the needs and preferences of older adults will be essential. Accordingly, involving middle-aged individuals, as future older adults, in the development and testing of digital health options might help ensure usability and effectiveness, supporting long-term health behaviors.

Future research with larger samples of older adults and different characteristics is warranted to help explore diverse perspectives and tailored needs of different aging populations. In particular, future studies could explore semi-automatized digital health programs for both nutrition and physical activity, focusing on delivering tailored dietary guidance, age-appropriate healthy recipes, and personalized physical exercises along with human support to enhance long-term engagement.

## Data Availability

The raw data supporting the conclusions of this article will be made available by the authors, without undue reservation.
