# Supplementary material for: Older adults' acceptability and perceived barriers to digital health tools integrating nutrition and physical activity: a focus group study
Source: Front Digit Health. 2026 Apr 22;8:1803847. doi: 10.3389/fdgth.2026.1803847 (PMC13144103; doi:10.3389/fdgth.2026.1803847)
Supplement: Supplementary file 1 [file Datasheet1.docx]

Supplementary Material

# Supplementary Data

**The research team developed a semi-structured interview guide. The guide consisted of open-ended prompts designed to explore participants’ experiences, perceptions, and expectations regarding digital health technologies related to nutrition and physical activity.**

**The guide included, but was not limited to, the following areas:**

- **Participants’ previous experiences with smartphone applications used to monitor diet and/or physical activity.
  Core prompts used: *“Have you ever used any applications or digital tools to monitor your physical activity? If yes, can you tell me about your experience with them? If no, could you tell me why you haven’t used them?”***

***“Have you ever used any applications or digital tools to monitor your diet or eating habits? If yes, how did you use them, and what was your experience? If no, what are the main reasons you have not used them?”***

- **Perceptions of useful features in digital health applications related to nutrition or physical activity.
  Core prompt used “*Thinking about an app related to nutrition or physical activity,***

***what characteristics or functions would it need to have for you to feel interested in using it? Are there any specific features that you would find particularly useful or important?”***

- **Attitudes toward reminders or notifications provided by health applications.
  Core prompt used: *“How would you feel about an app related to diet or physical activity sending you reminders or notifications? In what situations would these reminders be helpful for you, and when might they be annoying or unhelpful?”***

**These prompts served as a starting point to stimulate discussion but as typical in focus group research, participants were encouraged to interact with one another and expand on their experiences.**
